# Diabetes and Nonalcoholic Fatty Liver Disease

**DOI:** 10.1155/2012/404632

**Published:** 2011-12-14

**Authors:** Konstantinos Kantartzis, Amalia Gastaldelli, Faidon Magkos, Jean-Marc Lavoie

**Affiliations:** ^1^Division of Endocrinology and Diabetology, Angiology Nephrology, and Clinical Chemistry, Department of Internal Medicine IV, University of Tuebingen, Otfried-Müller Straße 10, D-72076 Tübingen, Germany; ^2^Stable Isotope Laboratory, Institute of Clinical Physiology, CNR, Via Moruzzi 1, 56100, Pisa, Italy; ^3^Devision of Geriatrics & Nutritional Science, Center for Human Nutrition, Washington University School of Medicine, 660 South Euclid Avenue, Campus Box 8031, Saint Louis, MO 63110, USA; ^4^Department of Kinesiology, University of Montreal, C.P. 6128, Succursale Centre Ville, Montreal, QC, Canada H3C 3J7

With almost every third individual affected in the general population in industrialized countries and increasing prevalence among children and adolescents, nonalcoholic fatty liver disease (NAFLD) represents the most common cause of chronic liver diseases such as cirrhosis, liver failure, and hepatocellular carcinoma and is therefore the most common cause of liver transplantation. In addition, in recent years NAFLD has emerged as a key player in human metabolism. Several studies demonstrate that NAFLD is strongly associated with insulin resistance and precedes the manifestation of type 2 diabetes and cardiovascular disease.

Of particular interest, the associations of ectopic fat accumulation in the liver with insulin resistance and type 2 diabetes are stronger than the respective of visceral and intramyocellular fat, implying that liver fat is an independent factor modifying the whole-body obesity-related metabolic risk. Thus, fatty liver may be not simply another manifestation of the metabolic syndrome, but it may itself induce or worsen insulin resistance and type 2 diabetes. In other words, fatty liver may be a determinant, not merely a marker of metabolic dysfunction. Though certainly, cause-and-effect relationships are hard to establish.

It is therefore why a concerted effort of the academic disciplines is requested to study the responsible mechanisms involved in the process of hepatic fat accumulation as well as the mechanisms regulating the crosstalk between fatty liver and other tissues important for regulation of metabolism in humans. In this special issue, we have invited some papers hoping to shed light on some aspects of this very interesting field.

In the first paper of this issue *“Diagnosis and evaluation of nonalcoholic fatty liver disease,”* epidemiology and tools for diagnosing NAFLD are reviewed. Regarding epidemiology, interesting data are provided on the interaction of NAFLD and type 2 diabetes, when concurrently present. In terms of diagnostic evaluation, of particular interest is the systematic presentation of old and novel biomarkers as well as panel markers (scores) and their sensitivity, specificity, positive and negative predictive values in estimating the amount of liver fat and differentiating more progressive forms of NAFLD, such as NASH and fibrosis, from simple steatosis.

The second paper of this issue *“Role of transcription factor modifications in the pathogenesis of insulin resistance,”* evaluates the diverse types of posttranslational modification of transcription factors in insulin-sensitive tissues and their putative role in the pathogenesis of insulin resistance. The authors particularly focused on the liver, where a lot of transcription factors have key roles in metabolic pathways critical for the pathogenesis of hepatic insulin resistance and NAFLD. For instance, forkhead box protein 1 (FOXO1) and cAMP response element binding protein (CREB) are major transcription factors for gluconeogenic gene expression, and sterol regulatory element-binding protein-1c (SREBP-1c) and carbohydrate response element-binding protein (ChREBP) are well known to regulate the expression of genes coding for lipogenic enzymes in hyperinsulinemic and hyperglycemic states. Thus, understanding the circumstances under which transcription factors undergo modifications will enhance our understanding of the molecular mechanisms leading to NAFLD and how it is related to insulin resistance.

The third paper of this issue *“Inhibition of aldose reductase activates hepatic peroxisome proliferators-activated receptor-*α* and ameliorates hepatosteatosis in diabetic db/db mice,”* examines the effect of inhibiting aldose reductase, the rate-limiting enzyme of the polyol pathway, on serum and hepatic triglyceride levels in db/db diabetic mice. The polyol-pathway is considered to be important in the development of a variety of diabetic complications but was thought not to play a significant role in liver disease, because the hepatic activity of aldose reductase is generally low. In this work, the authors inhibited aldose reductase, both pharmacologically and by a short-hairpin RNA, and found that this leads to a significant reduction in serum and hepatic triglycerides under hyperglycemia, possibly by activating PPAR*α* and thereby lipid oxidation. These findings indicate that the polyol pathway may be upregulated under certain conditions and may contribute to the development of NAFLD.

The fourth paper of this issue *“Cholesterol synthesis is associated with hepatic lipid content and dependent on fructose/glucose intake in healthy humans,”* reports on the association of liver and visceral fat with cholesterol homeostasis. Obesity, insulin resistance, and type 2 diabetes have been shown to independently correlate with increased endogenous cholesterol synthesis. However, the associations of body fat distribution with cholesterol metabolism have not been extensively studied. The authors found that visceral and liver fat are associated with cholesterol biosynthesis but not cholesterol intestinal absorption. This suggests that people with high liver fat and hypercholesterolemia will probably profit from statin treatment. The impact of high-fructose diet, which is thought to promote liver fat accumulation and hypercholesterolemia, on cholesterol homeostasis was also studied. Of interest, high-glucose diet appeared to stimulate cholesterol synthesis more than high-fructose diet did.

In the fifth article of this issue *“Predictors of impaired glucose regulation in patients with nonalcoholic fatty liver disease”*, the authors set out to find commonly measured demographic and laboratory parameters predicting an abnormal oral glucose tolerance test (OGTT) response (impaired glucose tolerance or diabetes mellitus) in patients with ultrasonography-diagnosed fatty liver. NAFLD is known to be closely associated with insulin resistance and type 2 diabetes; however, the characteristics of NAFLD patients having also hyperglycemia compared to NAFLD patients with normal glucose tolerance are not known. The authors found that if an OGTT is performed in patients with NAFLD and elevated liver enzymes, but no history of diabetes, almost half of them will display impaired glucose regulation, more likely those who are older, have higher BMI and lower HDL-cholesterol. Thus, at least those NAFLD patients with these characteristics should undergo further evaluation with an OGTT.

The final paper of this issue *“The role of metformin in the management of NAFLD”* is a review article summarizing the mechanism of action of metformin, the clinical studies performed so far on the use of metformin in patients with NAFLD, and the potential benefits of using metformin in NAFLD beyond its action in the liver. Currently, metformin is not considered to be an established treatment for NAFLD patients, probably because the existing clinical studies have been mostly small in sample size and short in duration and have provided in part controversial results. Most importantly, true randomized and controlled trials are lacking. Nevertheless, the high benefit-risk ratio of metformin, as well as its pleiotropic favourable effects (e.g., in promoting weight loss and lowering the risk of cancer), makes it an attractive treatment option for all patients with metabolic disturbances. Indeed, in this review the authors provide argumentation indicating that metformin may be of benefit in the treatment of both diabetic and nondiabetic patients with NAFLD.

The data presented and reviewed in this special issue highlight some novel or hitherto not elaborately studied aspects of NAFLD pathophysiology and treatment. Moreover, and perhaps more importantly, they underscore the complexity of factors and mechanisms regulating liver fat accumulation and depletion. We hope that they will encourage future efforts and research into understanding this very interesting and highly prevalent metabolic disorder.

*Konstantinos Kantartzis* *Konstantinos Kantartzis* *Amalia Gastaldelli* *Amalia Gastaldelli* *Faidon Magkos* *Faidon Magkos* *Jean-Marc Lavoie* *Jean-Marc Lavoie*

